# Trends and risk factors of bloodborne occupational exposure among healthcare workers in a Chinese tertiary hospital (2012–2022)

**DOI:** 10.3389/fpubh.2025.1619355

**Published:** 2025-10-08

**Authors:** Liyi Wang, Bing Gao, Shentai Li, Tianyuan Guo, Xuhua Cao, Mengsha Zhao, Yating Wang, Yan Liu

**Affiliations:** ^1^Second Hospital of Hebei Medical University, Shijiazhuang, China; ^2^Hebei Medical University, Shijiazhuang, Hebei, China

**Keywords:** COVID-19 pandemic, healthcare workers, long-term trends, occupational composition, bloodborne occupational exposure, professional title

## Abstract

**Objective:**

This study aimed to evaluate the epidemiological characteristics and long-term trends of bloodborne occupational exposure (BOE) among healthcare workers (HCWs) in a tertiary hospital in China from 2012 to 2022 (11 years) and evaluate BOE-associated factors during COVID-19 pandemic.

**Methods:**

A total of 1,725 self-reported cases of BOE were analyzed. The study comprised: (1) Descriptive analysis of demographic and professional variables; (2) Trend analysis of exposure events by season, month, sex, age, professional role, department, exposure source and occupational factors; and (3) Logistic regression analysis, with BOEs during the COVID-19 pandemic as the dependent variable.

**Results:**

BOEs were most prevalent among female, formally employed staff, nurses, 25-year-olds, those with 1–5 years of experience, and junior-title holders. High-risk settings included the neurosurgery department and wards; common exposure types were needlestick injuries (mostly to ungloved hands) and first-time exposures. Hepatitis B virus (HBV) was the primary exposure source, with most exposed individuals having a prior HBV vaccination history. Exposure frequency peaked in December and the fourth quarter of the year. (1) Longitudinal trends showed rising BOE incidence in December, spring, and among specific groups: females, 25-year-olds, hospital doctors (including postgraduate/doctoral trainees), nurses (including interns), and staff with 10–15 years of experience. Syphilis/suspected syphilis-related exposures also demonstrated an upward trend. (2) Logistic regression identified exposure month, occupation, length of service as independent factors associated with BOE during the COVID-19 pandemic (*p*<0.05).

**Conclusion:**

Targeted prevention and control strategies that focus on high-risk personnel, clinical departments, and specific procedures are essential to reduce the incidence of BOE among healthcare workers. Particular attention is required during public health emergencies (e.g., the COVID-19 pandemic), especially in addressing the January exposure peak, protecting physicians and mid-career staff with 16–20 years of service, and establishing cross-institutional mechanisms for coordinated BOE reporting and follow-up of support staff, in order to further minimize occupational risks. In addition, preventive measures such as targeted training programs, simulation-based exercises, and routine monitoring of HBV immunization status should be systematically implemented for trainees and newly recruited personnel.

## 1 Introduction

Bloodborne occupational exposure (BOE) represents a significant factor affecting the occupational health of healthcare workers (HCWs), contributing not only to physical harm but also to psychological distress, including fear, anxiety, and depression, as well as financial burden (1–3). Our early survey confirmed significant economic burden of BOE, with average management costs and reexamination rates (0.00–63.64%) varying substantially by exposure source (4). Another report documented the average management cost of bloodborne BOEs as follows: hepatitis B virus (HBV) (Chinese Yuan [RMB] 5,936/USD 897), hepatitis C virus (HCV) (RMB 5,738/USD 867), *Treponema pallidum* (TP) (RMB 4,508/USD 681), human immunodeficiency virus (HIV) (RMB 12,709/USD 1,920), and needlestick injury of unknown source (RMB 7,441/USD 1,124) (5).

Globally, the scale of the issue remains alarming. According to a 2023 report by the U. S. Occupational Safety and Health Administration (OSHA), approximately 5.6 million HCWs face infection risks from sharps injuries (6). An estimated 78% of emergency healthcare personnel have experienced more than one needlestick injury (7, 8). Without intervention, the risk of transmission of serious infectious diseases such as HBV through BOE can be as high as 30% (9).

Despite this need, most existing BOE surveillance studies are limited by short monitoring periods (typically 3–5 years).and lacking long-term trend analysis (≥10 years) to identify persistent high-risk patterns. And our manuscript can fill this international gap. Additionally, research on how COVID-19 reshaped BOE risks, especially in large Chinese tertiary hospitals (key pandemic response sites), remains fragmented. This gap is critical: hospitals like ours faced unique pandemic challenges (large-scale staff deployment for external COVID-19 response, altered clinical workflows, shifting workloads) that may have modified BOE risks but are understudied. To address this, we conducted this study, aiming to provide empirical evidence for stratified, context-specific BOE prevention for both routine and public health emergency scenarios.

## 2 Participants and methods

### 2.1 Participants

This study included HCWs from the Second Hospital of Hebei Medical University who experienced BOE between January 2012 and December 2022 and completed a *BOE Registration Form* (hereafter referred to as the reporting form).

This study was conducted at the Second Hospital of Hebei Medical University, a comprehensive medical center integrating healthcare, teaching, research, prevention, rehabilitation, and emergency services. The hospital currently operates across four campuses (i.e., Main Campus, Luquan Campus, North Campus, and Shangzhuang Campus) with the Zhengding Campus under construction. It has 2,816 approved beds and 4,574 open beds, with a total staff of 6,294. The hospital also trains 1,060 undergraduate students and 1,340 postgraduate students (master’s and doctoral). During the COVID-19 pandemic, as a designated provincial treatment center, the hospital deployed a large number of healthcare workers to support medical efforts in other provinces and institutions.

Ethical approval for this study was granted by the Ethics Committee of the Second Hospital of Hebei Medical University (Approval number: 2020-R522 and 2024-R406).

### 2.2 Methods

#### 2.2.1 Survey method

A retrospective analysis was conducted based on reported cases of BOE (10), defined as the state in which workers, during occupational activities, are exposed to blood or other potentially infectious materials containing bloodborne pathogens (microorganisms present in blood and certain body fluids that can cause human disease) through the eyes, mouth, nose, other mucous membranes, damaged skin, or parenteral routes.

##### 2.2.1.1 Inclusion criteria

HCWs were included if they met all of the following conditions: (i) experienced BOE between January 1, 2012 and December 31, 2022; (ii) the exposure occurred within the study hospital; (iii) the incident was reported to the infection control department; and (iv) a completed BOE registration form was submitted.

##### 2.2.1.2 Exclusion criteria

Cases were excluded if any of the following applied: (i) failure to report the incident to the Infection Control Department via telephone; (ii) failure to complete the BOE Registration Form; (iii) missing or incomplete data.

##### 2.2.1.3 Handling of missing data

(i) Immediate completion at the time of reporting: After a BOE event, healthcare workers were required to report in person to the Infection Control Department and complete the Occupational Exposure Information Registration Form. Infection control staff verified the entries on site and immediately prompted for clarification or completion of any missing or ambiguous information (e.g., “uncertain whether hepatitis B vaccination was received”). Only fully completed paper forms were accepted and archived.
(ii) Dedicated data entry with double verification: Archived paper forms were independently entered into the electronic database by two trained infection control staff using a dual-entry method. Upon completion, entries were cross-checked automatically in SPSS. In cases of discrepancies (e.g., “years of service” recorded as “3 years” vs. “5 years”), the original paper form was traced to verify and correct the record, ensuring complete consistency between electronic and paper data.
(iii) Three-tier quality control with periodic follow-up: Telephone follow-up was conducted after each BOE incident both to monitor post-exposure management and to re-verify registration form data. Any omissions identified in the original records were immediately corrected in both paper and electronic files. This three-level quality control and periodic follow-up process ensured prevention of data loss from the source.

#### 2.2.2 Emergency treatment and follow-up

BOE was categorized as either needlestick injury or exposures involving the skin and mucous membranes. In the event of a needlestick injury, initial wound management involved expressing blood from the proximal to the distal end, followed by thorough rinsing with clean water and disinfection. For skin exposure, the affected area was rinsed with running water. In cases involving mucous membrane exposure, the exposed area was repeatedly flushed with sterile normal saline.

Follow-up procedures protocols were implemented based on the pathogen associated with the exposure. The duration and specific components of follow-up care were determined by the incubation period and clinical characteristics of the implicated pathogen.

#### 2.2.3 COVID-19 period definition

Based on the adjustment milestones of China’s COVID-19 prevention and control policies and the data collection timeframe of this study, the observation periods were clearly divided as follows:

(i) COVID-19 pandemic period (December 2019–December 2022): According to the Law of the People’s Republic of China on the Prevention and Treatment of Infectious Diseases (11), infectious diseases are classified into Categories A, B, and C, with Category A requiring the most stringent mandatory measures (e.g., isolation, regional lockdown). COVID-19 was first reported in China in late 2019, and beginning January 20, 2020, it was temporarily managed under the “Category B management with Category A measures” (“B type with A management”) policy. The pandemic period was defined as extending from this point until December 2022, when comprehensive optimization of prevention and control measures was implemented. From January 8, 2023, COVID-19 management was downgraded to routine Category B measures (“B type with B management”).
(ii) Non-pandemic period (January 2012–November 30, 2019): A stable pre-pandemic phase unaffected by COVID-19.

### 2.3 Statistical analysis

#### 2.3.1 The validated dataset was then imported into SPSS version 22.0 for statistical analysis

Categorical data were expressed as percentages, and comparisons between groups were conducted using the chi-squared (*χ^2^*) test. A two-sided *p*-value of less than 0.05 was considered statistically significant.

For continuous variables with a normal distribution, comparisons between groups were performed using analysis of variance (ANOVA). For data not conforming to normal distribution, comparisons between groups were performed using the rank sum test.

#### 2.3.2 Logistic regression analysis

The objective was to identify independent factors associated with BOE during the COVID-19 pandemic. The dependent variable was whether the exposure occurred during the COVID-19 pandemic period (binary: 1 = pandemic period, December 2019–December 2022; 0 = non-pandemic period, January 2012–November 2019). Independent variables were selected based on preliminary univariate analysis (χ^2^ test), and only those with statistically significant group differences (*p* < 0.05) were included. Binary logistic regression (Backward: Conditional) was then performed to determine independent risk factors, with statistical significance set at *p* < 0.05. Variable coding is presented in Table 1.

**Table 1:** Composition of bloodborne occupational exposure sources.

Year	HBV	Needlestick injury of unknown source	Non-bloodborne pathogens	HCV	Syphilis + suspected syphilis	HIV + suspected HIV	HBV + HCV	Tetanus	Syphilis + AIDS	Syphilis + HBV	Pulmonary tuberculosis	Hepatitis C + syphilis	Rabies virus	HBV, HIV	Others	Total
2012	52	13	0	2	2	0	1	0	0	0	0	0	2	0	1	73
2013	54	26	0	1	1	1	2	0	0	0	0	0	0	0	1	86
2014	56	43	0	9	0	1	1	2	0	0	1	0	0	0	0	113
2015	76	37	8	6	2	5	2	0	0	0	1	1	1	2	1	142
2016	84	33	18	20	23	1	1	0	3	1	0	1	0	0	0	185
2017	79	77	30	12	6	6	1	0	0	1	0	0	0	0	0	212
2018	74	62	57	22	14	7	0	1	0	1	0	0	0	0	0	238
2019	96	49	39	19	16	7	1	1	0	0	1	1	0	0	1	231
2020	47	23	34	11	5	2	3	2	0	1	0	1	0	0	2	131
2021	66	32	43	11	6	2	0	2	1	0	0	0	0	0	0	163
2022	45	23	52	4	15	4	0	2	1	0	0	0	0	0	5	151
Total	729	418	281	117	90	36	12	10	5	4	3	4	3	2	11	1725

## 3 Results

### 3.1 Temporal distribution of cumulative BOE cases

#### 3.1.1 Annual distribution

**Figure 1:**
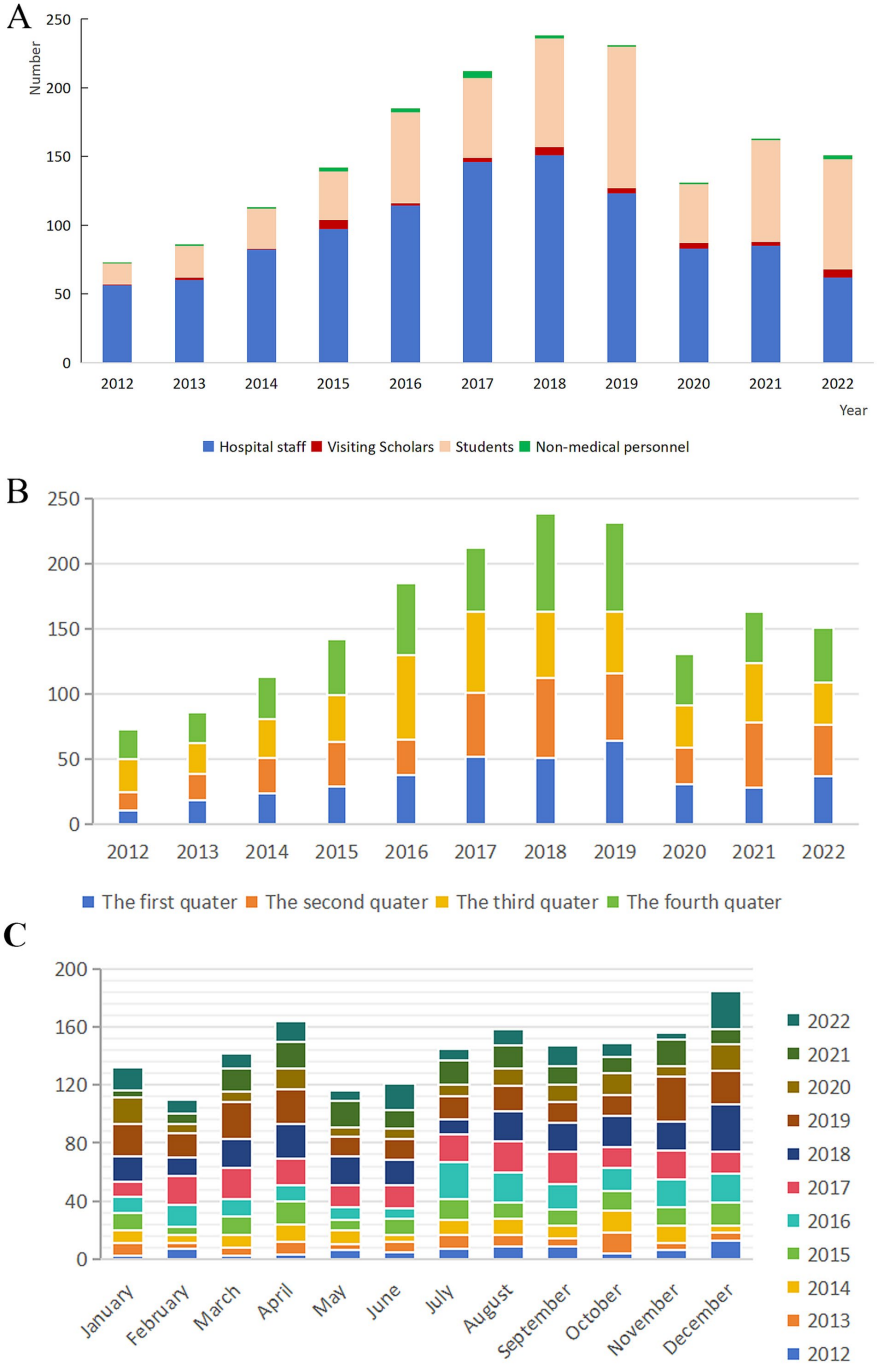
Long-term trends in actively reported BOE cases, 2012–2022. **(A)** Annual trend in reported BOE cases. **(B)** Seasonal distribution of reported cases. **(C)** Monthly distribution of reported cases.

As shown in Supplementary Table 1, from January 2012 to December 2022, a total of 1,725 HCWs reported BOE, which regular staff accounted for the most (1,059 cases, 61.39%). As illustrated in Figure 1A, the cumulative number of self-reported exposure cases exhibited an upward trend from 2012, peaking in 2018, followed by a consistent decline from 2019 onwards.

Regarding seasonal and monthly distribution (Figures 1B,C), the fourth quarter accounted for the highest cumulative case (490 cases, 28.40%). Longitudinal analysis of seasonal trends from 2012 to 2022 revealed a general increase in fourth-quarter exposure cases until 2018. However, this upward trend was interrupted by a decline in 2017, indicating a temporary deviation from the overall pattern. December was consistently the month with the highest exposure frequency (185 cases, 10.72%). Longitudinal analysis revealed that fourth-quarter cases generally increased until 2018, with a temporary decline in 2017.

### 3.2 Characteristics of the exposed population

**Figure 2:**
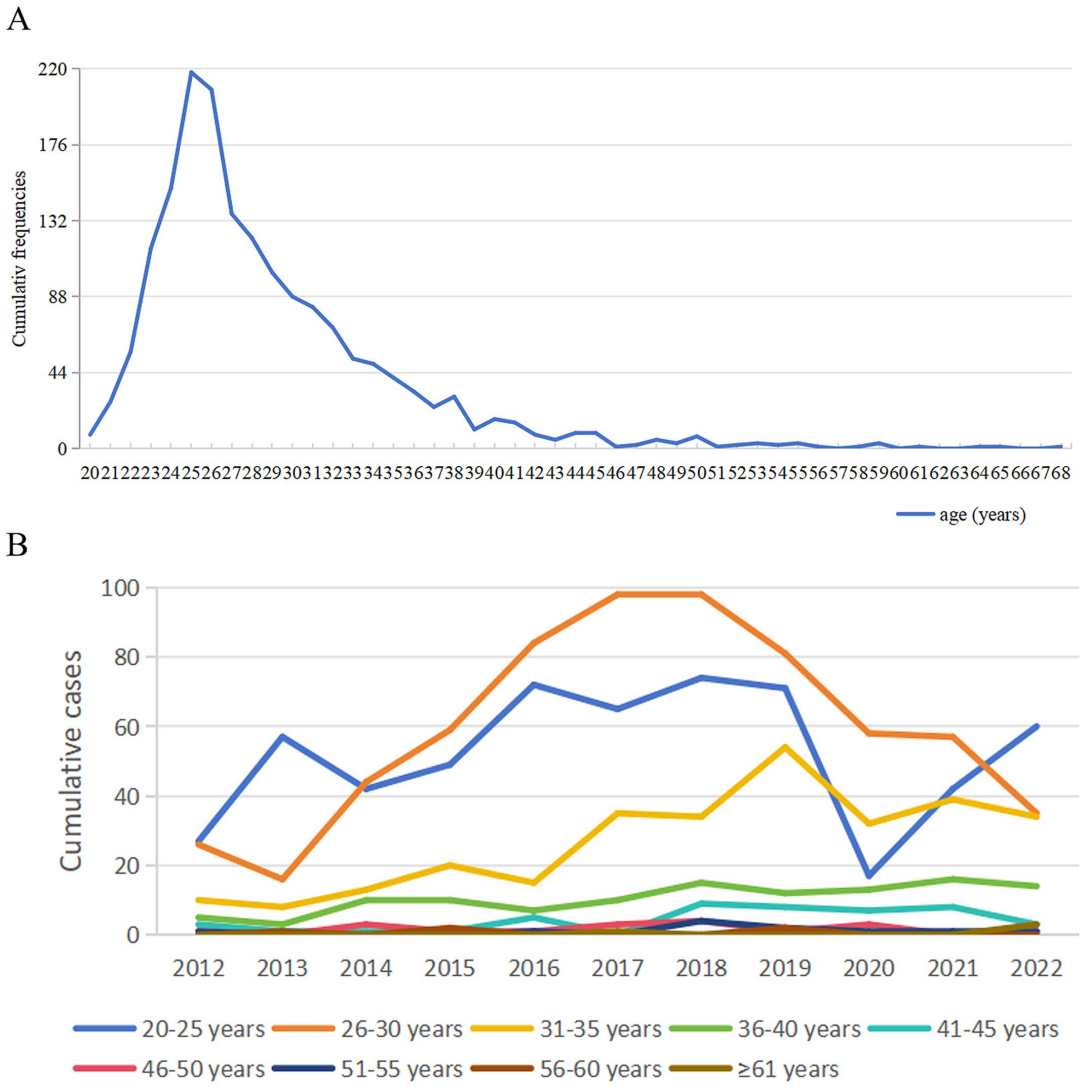
Long-term trends in BOE cases by age, 2012–2022. **(A)** Cumulative number of cases by age group. **(B)** Cumulative exposure incidents by age group. ys, years.

The majority of bloodborne occupational exposures (BOEs) occurred among female healthcare workers, who accounted for 76.29% of the total cases (1,316 individuals; Table 2). In terms of age, the exposed population exhibited a relatively young profile, with a median age of 27 years (interquartile range: 25–31) and an overall range from 20 to 68 years. Notably, individuals aged 25 experienced the highest cumulative incidence. A marked decline in case numbers was observed between the ages of 26 and 37, while the 20–25 age group showed a sharp increase in exposures following 2020 (Figure 2).

**Table 2:** Sex composition of reported occupational exposure cases, 2021-2022.

Sex	Male		Female	
year	Number	Percentage (%)	Number	Percentage (%)
2012	11	15.07	62	84.93
2013	9	10.47	77	89.53
2014	19	16.81	94	83.19
2015	29	20.42	113	79.58
2016	35	18.92	150	81.08
2017	43	20.28	169	79.72
2018	60	25.21	178	74.79
2019	74	32.03	157	67.97
2020	33	25.19	98	74.81
2021	49	30.06	114	69.94
2022	47	31.13	104	68.87
Total	409	23.71	1316	76.29

**Figure 3:**
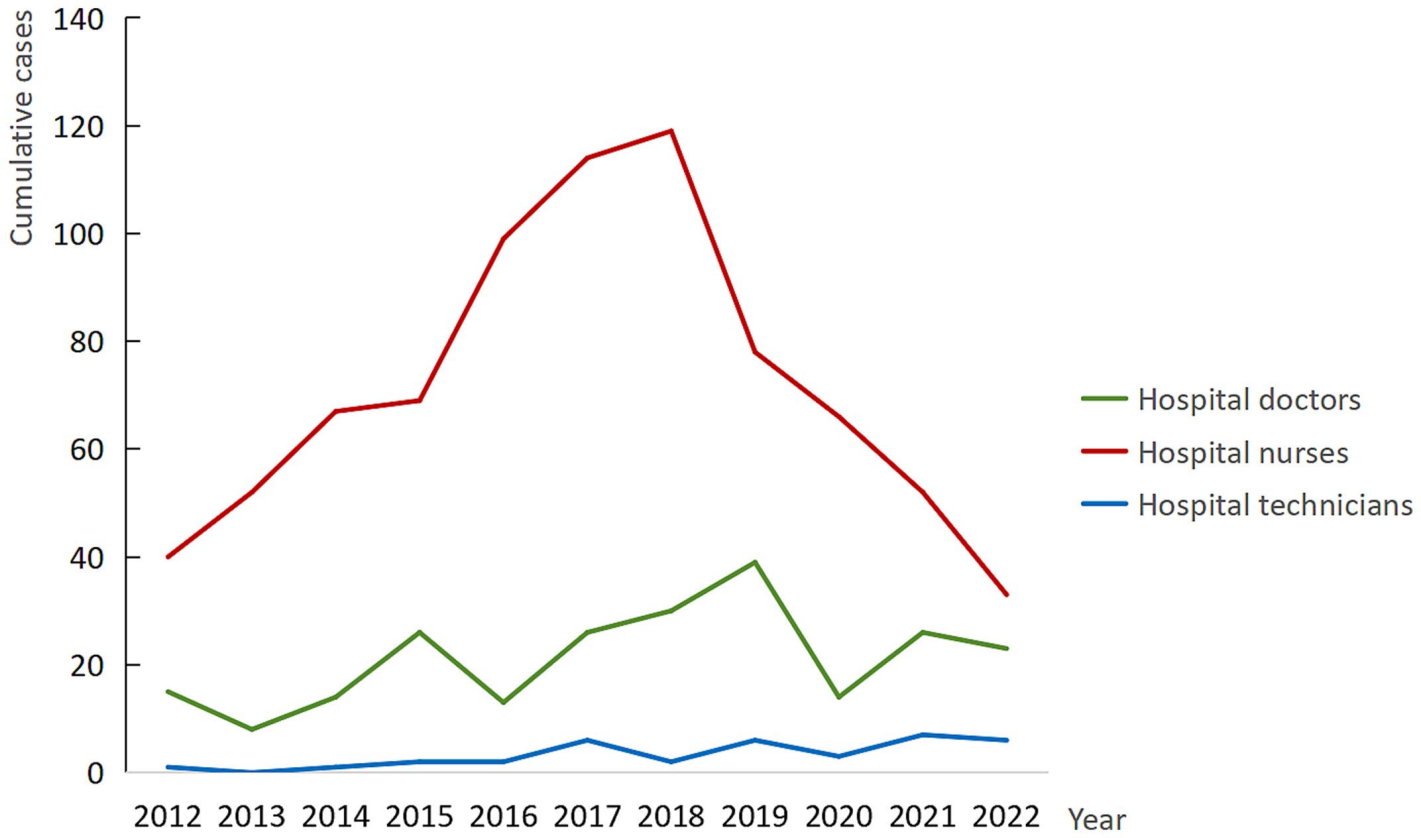
Long-term trends in BOE by staff composition in the study hospital, 2012–2022.

Occupational analysis revealed that nurses constituted the largest proportion of reported BOEs, representing 53.22% of all cases (918 individuals). However, this predominance was not consistent across all years. In 2019, 2021, and 2022, doctors reported a higher number of exposures than nurses, indicating a shift in occupational risk patterns during these periods. This variation in occupational composition was statistically significant (χ^2^ = 62.91, *p* < 0.001), with detailed regression results provided in Supplementary Table 1. As illustrated in Figures 3, a notable decline in cumulative BOE cases among nurses began in 2018, with a continued downward trend observed through 2022.

With respect to length of service, individuals with 1–5 years of experience accounted for the largest share of exposures (422 cases, 38.62%), particularly dominating the case distribution from 2014 to 2018. However, from 2019 onward, healthcare workers with 5–10 years of service emerged as the most affected group, suggesting a shift in exposure risk associated with professional tenure (Table 3).

**Table 3:** Distribution of occupational exposure cases by length of service, 2021–2022.

Identity	Working years	2012	2013	2014	2015	2016	2017	2018	2019	2020	2021	2022	Total
Regular staff	≤1 year	20	27	8	11	21	13	15	5	1	9	3	133
	1–5 years	17	19	44	57	61	72	63	39	22	15	13	422
	5–10 years	5	6	11	19	20	47	46	57	36	42	23	312
	10–15 years	6	2	8	2	5	6	6	8	11	13	21	88
	15–20 years	4	6	9	5	5	7	9	4	4	1	0	54
	>20 years	3	0	2	3	2	1	12	10	9	5	2	49
	Total	56	60	82	97	114	146	151	123	83	85	62	1,058
Students	Students	15	23	29	35	66	58	79	103	43	74	80	605
Visiting staff	Visiting staff	1	2	1	7	2	3	6	4	4	3	6	39
Non-medical personnel	Non-medical personnel	1	1	1	3	3	5	2	1	1	1	3	22
Total		73	86	113	142	185	212	238	231	131	163	151	1725

### 3.3 High-risk departmental and location distribution

Neurosurgery, cardiology, gastroenterology, emergency, and neurology accounted for the greatest share of reported exposures, contributing 142 (8.23%), 124 (7.19%), 93 (5.39%), 88 (5.10%), and 84 (4.87%) cases, respectively (Table 4). When mapped to physical space, 10 distinct locations were identified, of which inpatient wards predominated, generating 980 events (56.78%), while operating rooms supplied a further 361 (20.92%).

**Table 4:** Departmental distribution and exposure-methods-distribution.

Item	Category	Cumulative cases, n (%)		Proportion (%)
Exposure location	Ward	980		56.81
	Operating room	361		20.93
	Emergency department	130		7.54
	ICU	100		5.80
	Medical/technical examination departments	95		5.51
	Outpatient clinic	38		2.20
	Laboratory	9		0.52
	Interventional catheterization room	5		0.29
	Temporary medical waste storage area	5		0.29
	Other	2		0.11
Mode of exposure	Needlestick injury	Disposable syringe needle	403	23.36
		Suture needle	186	10.78
		Arterial blood gas needle	125	7.25
		Scalp needle	101	5.86
		Insulin needle	84	4.87
		Indwelling needle	76	4.41
		Phlebotomy needle	68	3.94
		Puncture needles (bone marrow, lumbar puncture, etc.)	46	2.67
		Infusion needle	34	1.97
		Discarded needle	21	1.22
		Intradermal test needle	9	0.52
		Radiofrequency needle	8	0.46
		Other types of needles	70	4.06
		Subtotal	1,231	71.36
	Splash of blood, body fluids, or secretions		229	13.28
	Injury by surgical instruments		142	8.23
	Injury by glass products		47	2.72
	Injury by unidentified sharp objects		16	0.93
	Forceps		5	0.29
	Unidentified wound discovered after procedure		5	0.29
	Electrode		4	0.23
	Protective clothing		3	0.17
	Other		3	0.17
	Bite by patient’s teeth		3	0.17
	Scratch		3	0.17
	Sheet metal/wire		3	0.17
	Instrument basket		2	0.12
	Sharp instrument		2	0.12
	Other		27	1.57
First exposure	Yes		1,412	81.86
	No		313	18.14

### 3.4 Exposure-related characteristics

Table 4 summarizes the instruments and circumstances underlying each event. More than four-fifths of the cohort (1,412; 81.86%) were experiencing their first BOE, and needlestick injuries dominated the etiological profile, representing 1,231 incidents (71.36%). Disposable syringe needles were implicated in 403 of these punctures (32.74% of all needlestick injuries). The remaining exposures arose from direct contact with blood or body fluids (229; 13.27%) and from injuries inflicted by surgical instruments (142; 8.23%). As shown in Table 5, the hand was the anatomical site affected in 1554 reports (90.09%), with 1,550 involving the hand alone and four additional cases combining hand and eye contamination; glove use was documented in fewer than one-fifth of these episodes (19.05%).

**Table 5:** Distribution of exposure sites.

Year	Hand	Eye mucosa	Foot	Leg	Arm	Oral mucosa	Face	Hand + eye	Skin	Knee	Back	Other sites	Total
2012	70	1	1	0	1	0	0	0	0	0	0	0	73
2013	79	2	2	0	1	0	0	2	0	0	0	0	86
2014	100	3	3	2	1	3	0	0	0	0	1	0	113
2015	131	4	3	2	1	0	0	0	1	0	0	0	142
2016	170	10	2	1	0	1	0	1	0	0	0	0	185
2017	193	8	7	1	1	0	1	1	0	0	0	0	212
2018	203	17	4	0	9	0	2	0	2	1	0	0	238
2019	213	14	1	1	1	0	0	0	0	0	0	1	231
2020	116	9	2	1	1	1	0	0	0	1	0	0	131
2021	146	14	3	0	0	0	0	0	0	0	0	0	163
2022	129	11	3	1	0	1	3	0	0	0	1	2	151
Total	1,550	93	31	9	16	6	6	4	3	2	2	3	1725

### 3.5 Exposure sources and HBV prevention status

#### 3.5.1 Exposure source distribution

As shown in Table 1, the three most common sources of BOE cases were HBV (729 cases, 42.26%), needlestick injuries from unknown sources (no identifiable pathogen, 418 cases, 24.23%), and non-bloodborne pathogens (281 cases, 16.29%). HBV was the leading source of exposure from 2012 to 2021. However, by 2022, the number of non-bloodborne pathogens surpassed HBV. Syphilis/suspected syphilis-related exposures also demonstrated an overall upward trend.

#### 3.5.2 HBV vaccination and post-exposure management

As shown in Table 6, among the 749 cases involving BOE to HBV (including co-exposures to other pathogens), 670 cases (89.45%) had previously received hepatitis B vaccination (only 83.43% received one hepatitis B vaccine dose), 40 cases had been administered hepatitis B immunoglobulin (HBIG) and 731 cases (97.60%) implemented immediate emergency measures (only 76.47% followed standard protocols).

**Table 6:** Characteristics of 749 healthcare workers with occupational exposure to HBV.

Item	Number (cases)			
Number of exposures	First time			655
	2 times			71
	3 times			4
	4 times			2
	Multiple times			17
Previously received HBV vaccine injection	No			62
	Yes	1 time	559	670
		2 times	66	
		3 times	37	
		4 times	3	
		5 times	1	
		6 times	3	
		8 times	1	
	Unknown			17
Previously received HBIG injection	No			687
	Yes			40
	Unknown			22
Emergency handling after exposure	No			18
	Yes	Qualified handling	559	731
		Unqualified handling	172	

### 3.6 COVID-19 pandemic-related BOE analysis

#### 3.6.1 Definition of pandemic and non-pandemic periods

The COVID-19 pandemic period was defined as December 2019–December 2022, and the non-pandemic period as January 2012–November 2019.

#### 3.6.2 Group comparison of potential influencing factors

Group comparisons were conducted using the χ^2^ test to examine differences between the pandemic and non-pandemic groups across nine potential influencing factors. Significant differences were observed in the distributions of exposure month, sex, occupation, professional title, length of service, number of exposures, history of hepatitis B vaccination, and history of HBIG administration (all *p* < 0.05). These variables with significant group differences were subsequently included as independent variables in the binary logistic regression analysis. Variable coding is presented in Table 7.

**Table 7:** Comparison between pandemic and non-pandemic groups.

Item	Factors	Non-pandemic groups	Pandemic groups	Wald X^2^	*P*
Exposure quarter	First	288	96	4.124	0.248
	Second	284	117		
	Third	337	111		
	Fourth	348	144		
Exposure month	January	93	39	33.236	0.000
	February	87	23		
	March	108	34		
	April	117	47		
	May	84	32		
	June	83	38		
	July	112	33		
	August	119	39		
	September	108	39		
	October	113	36		
	November	126	30		
	December	107	78		
Sex	Male	270	139	12.743	0.000
	Female	987	329		
Occupation	Doctor	495	249	34.522	0.000
	Nurse	721	197		
	Physician	24	17		
	Others	17	5		
Professional title	Junior	874	315	50.091	0.000
	Intermediate	248	136		
	Associate Senior	27	11		
	Senior	12	1		
	No title	96	5		
Length of service	<1 year	121	13	131.241	0.000
	1–5 years	367	52		
	6–10 years	202	113		
	11–15 years	42	46		
	16–20 years	49	5		
	>20 years	33	16		
	Students	400	205		
	Non-medical workers	17	5		
	Trainees	26	13		
BOE times	First time	1,024	388	48.189	0.000
	Second time	126	65		
	The third time	7	0		
	The fourth time	2	0		
	Many times	38	15		
	Unknown	60	0		
History of hepatitis B vaccine injection	Yes	1,103	451	28.464	0.000
	No	108	11		
	Unknown	46	6		
History of HBIG injection	Yes	63	9	20.223	0.000
	No	1,143	455		
	Unknown	51	4		

#### 3.6.3 Logistic regression analysis of independent factors for BOE during the pandemic

A binary logistic regression was conducted using the COVID-19 pandemic period (versus the non-pandemic period) as the dependent variable and identified three independent factors associated with BOE during the pandemic (*p* < 0.05; Table 8). Logistic regression analysis revealed that, compared with January, the risk of BOE was significantly lower in all other months except July, with December showing the lowest risk (OR = 0.227, 95% CI: 0.132–0.390, *p* < 0.05). Regarding occupation, technicians had a significantly lower risk of BOE compared with doctors (OR = 0.395, 95% CI: 0.191–0.819, *p* < 0.05). In terms of length of service, employees with 1–5 years (OR = 0.343, 95% CI: 0.135–0.873) and 6–10 years (OR = 0.392, 95% CI: 0.180–0.852) of service had significantly reduced risks compared with those with less than 1 year, whereas those with 16–20 years of service had a markedly increased risk (OR = 3.674, 95% CI: 1.456–9.272, *p* < 0.05).

**Table 8:** Logistic regression analysis of factors associated with occupational exposure during the COVID-19 pandemic period.

Independent risk factors		b	Sb	Wald χ^2^	*P*	OR	95%CI
Exposure month				44.471	0.000		
	February vs. January	−0.745	0.269	7.649	0.006	0.475	0.28–0.805
	March vs. January	−0.941	0.307	9.384	0.002	0.390	0.214–0.713
	April vs. January	−1.133	0.275	16.937	0.000	0.322	0.188–0.553
	May vs. January	−0.753	0.254	8.805	0.003	0.471	0.287–0.775
	June vs. January	−0.887	0.284	9.732	0.002	0.412	0.236–0.719
	August vs. January	−1.264	0.273	21.398	0.000	0.282	0.165–0.483
	September vs. January	−1.086	0.261	17.277	0.000	0.338	0.202–0.563
	October vs. January	−0.867	0.265	10.683	0.001	0.420	0.25–0.707
	November vs. January	−1.076	0.271	15.790	0.000	0.341	0.201–0.58
	December vs. January	−1.483	0.276	28.852	0.000	0.227	0.132–0.390
Occupation				17.574	0.000		
	Technician vs. doctor	−0.928	0.371	6.245	0.012	0.395	0.191–0.819
Length of service				106.734	0.000		
	1–5 years vs. <1 year	−1.070	0.476	5.040	0.025	0.343	0.135–0.873
	6–10 years vs. <1 year	−0.938	0.397	5.587	0.018	0.392	0.180–0.852
	16–20 years vs. <1 year	1.301	0.472	7.588	0.006	3.674	1.456–9.272
Constant		−22.242	4404.064	0.000	0.996	0.000	

## 4 Discussion

In the early 20th century, China successively introduced regulatory frameworks such as the *Regulations on Hospital Infection Management* (12), *Guidelines on BOE Protection of HCWs to HIV (Trial)* (13), and *Guidelines for BOE Protection to Bloodborne Pathogens* (10), establishing standardized protocols for BOE management and protection nationwide. This heightened awareness likely contributed to the rising trend in actively reported BOE cases from 2012 to 2018 in this study. This 11-year longitudinal analysis addresses key gaps in existing research, while critical reflection on its limitations and targeted interpretation of trends provide actionable insights for BOE prevention.

### 4.1 Interpretation of Core epidemiological trends

The BOE characteristics identified in this study are generally consistent with findings from prior cross-sectional studies (14–24). Our study’s long-term data reveals three overarching trends that demand targeted intervention, with interpretations focused on why these patterns emerged rather than restating numerical results:

First, persistent high-risk groups (females, 25-year-olds, early-career staff, and interns) reflect structural and experiential factors. The overlap between the 25-year-old age group and 1–5 years of service aligns with a “competence gap”: HCWs at this stage take on independent operational roles but lack sufficient proficiency, increasing error rates during sharp handling or patient care. Interns face compounded risks due to limited clinical experience, inadequate risk awareness, and psychological stress (e.g., nervousness during procedures) (25, 26), a vulnerability often underemphasized in short-term studies (24). Thus, regarding the observed increase in BOE risk among personnel with 10–15 years of service, it should be noted that most short-term studies suggest that BOE risk decreases with increasing length of service (24). However, our 11-year longitudinal data indicate a rebound in risk within the 10–15 year service group.

Second, seasonal and monthly peaks (December and spring) are tied to healthcare system dynamics. The December surge correlates with pre-Spring Festival patient discharge pressures, which increase HCW fatigue and reduce adherence to sharp disposal protocols (e.g., improper needle placement in puncture-proof containers). Spring peaks stem from post-holiday staffing shortages (exacerbated by county-level hospital closures during the festival) and concentrated admissions of critically ill patients, leading to “expedited care” that skips protective steps (e.g., glove use).

Third, department-specific risks (neurosurgery, inpatient wards) highlight procedure-related hazards. Neurosurgery’s high exposure rate is not merely a numerical trend but a function of its unique workflow: diverse sharp instruments, limited operating space, and higher patient agitation rates increase accidental punctures or spills, consistent with surgical specialty risk profiles (18) but with nuances (e.g., instrument diversity) that prior cross-sectional studies (22) did not fully explore.

### 4.2 Implications of the COVID-19 pandemic for blood-borne occupational-exposure prevention and control

The COVID-19 emergency (December 2019–December 2022) reshaped the epidemiology of BOEs in ways that extend beyond the immediate virological threat. By focusing on a large tertiary hospital designated as a provincial COVID-19 hub, the present analysis offers one of the first empirical accounts of how a public-health crisis reconfigures sharps-related risk in the Chinese setting.

A conspicuous downward inflection in facility-reported exposures after 2019 was not indicative of safer practice, but of workforce redistribution. Large contingents of personnel were seconded to off-site response teams; incidents occurring in these external units were logged by the host institution rather than by our infection-control office. The artefactual decline underscores the need for multi-center surveillance protocols that track mobile staff longitudinally and consolidate exposure data across administrative boundaries.

Logistic modeling identified three pandemic-specific determinants that remained significant after adjustment for background demographics and departmental caseload. First, exposures clustered in January, a pattern attributable to Spring-Festival Rota changes that temporarily replaced experienced staff with less-seasoned clinicians. The finding implies that holiday-transition “emergency safeguards.” For example, mandated dual verification of sharps procedures should be embedded in future surge plans. Second, physicians rather than nurses emerged as the occupational group at highest risk (14, 17), a reversal of the pre-pandemic hierarchy. The elevation coincided with the expanded use of aerosol-generating and invasive interventions such as tracheal intubation in COVID-19 units, and it signals the urgency of role-specific protective devices (e.g., safety-engineered syringes) for front-line doctors and intensivists during infectious-disease emergencies. Third, employees with 16–20 years of service experienced an unanticipated excess risk. Senior clinicians were frequently redeployed to surge wards or asked to lead rapid-response teams, thereby encountering high-risk scenarios from which they had previously been shielded. The observation challenges the conventional view that experience is invariably protective and argues for the inclusion of senior staff in crisis-oriented refresher training and personal-protective-equipment fit-testing.

Collectively, these results demonstrate that pandemic conditions do not merely amplify baseline exposure frequencies; they re-order the entire risk architecture. Incorporating crisis-specific variables into occupational-surveillance systems will be essential for anticipating, rather than merely reacting to, the distributional shifts that accompany the next public-health emergency.

### 4.3 Targeted preventive strategies

To translate the observed epidemiological signals into durable protection, interventions are grouped along three mutually reinforcing axes: high-risk cohorts, resource optimization, and emergency preparedness, each anchored in pre-employment training and continuously audited competence.

Addressing high-risk groups. Interns and other novices experience the steepest learning curve exactly when their procedural exposure peaks; consequently, simulation-based modules that rehearse safe handling of safety-engineered syringes, winged-steel sets and surgical sharps are now prerequisite to ward placement and are examined items in the end-of-rotation assessment. Conversely, clinicians in their sixteenth to twentieth service year, unexpectedly over-represented during COVID-19 require crisis-specific refreshers that couple leadership responsibilities with just-in-time protocols for surge wards, high-flow aerosol procedures and makeshift intensive-care areas.

Optimizing protective resources. Because gloved hands nevertheless sustained nine in 10 injuries, the 2022 pilot that introduced size-XS gloves and a personalized ordering portal is being scaled hospital-wide to eliminate fit-related non-compliance. Hepatitis B protection is completed through an obligatory pre-employment serological screen, full vaccine coverage under the employee health-insurance umbrella, and algorithm-driven reminders for booster doses whenever anti-HBs titers fall below 10 IU L^−1^, closing the gap left by the residual 10.55% of vaccine-naïve, exposure-prone staff (27).

Strengthening emergency preparedness. The artificial drop in facility-reported exposures during COVID-19 underscores the need for a provincial, or preferably national, registry that follows every seconded health-care worker so that no event is lost to administrative fragmentation. Seasonal staffing transitions are equally critical: just-in-time drills scheduled each November (pre-December caseload ascent) and February (post-Spring Festival workforce churn) entrench protocol adherence when experiential memory and supervisory density are lowest.

### 4.4 Methodological limitations

The interpretive weight of any longitudinal BOE audit rests on the fidelity of its numerator. Because reporting remains voluntary, the database inevitably under-represents trivial splashes or fleeting needle scratches that busy clinicians consider too minor to disclose. The pandemic amplified this deficit: staff seconded to off-site COVID-19 units reported incidents to the host facility, producing an artefactual trough in our 2020–2022 trend line. Complementing passive metrics (weights of sharps containers, barcode-triggered disposal audits, or electronic health-record triggers) will be required to quantify the true shortfall.

Single-center tertiary care confers a second, complementary limitation. The hospital’s multi-campus infrastructure, high-acuity referral mix and dedicated surge teams are not mirrored in district hospitals or primary-care clinics where staffing ratios, procedural complexity and PPE availability diverge. Multi-center consortia that stratify results by level of care are therefore needed before exporting risk estimates.

Within the multivariable model itself, sparse data in some experience strata widened confidence intervals—most notably for the 16–20-year service group that the pandemic unexpectedly elevated. Although the direction of effect was consistent across sensitivity analyses, the magnitude should be regarded as provisional until larger samples or pooled datasets stabilize the estimates.

Residual confounding is probable. Cumulative patient load per clinician, minute-by-minute workload pressure, rapid transitions to telemedicine and subtle batch-to-batch variations in glove thickness or syringe design could all mediate exposure propensity but were not captured. Finally, formal diagnostics for log-linearity, link specification and influential outliers were omitted because the analysis was intended as hypothesis-generating surveillance rather than confirmatory causal inference; subsequent work that aspires to policy significance should embed comprehensive goodness-of-fit testing.

### 4.5 Contributions to the field

By spanning 11 consecutive years the cohort transcends the customary 3–5 year horizon (21, 22), revealing career-cycle phenomena, such as the late-career risk resurgence among mid-senior clinicians that shorter windows overlook. To our knowledge this is the first uninterrupted decade-long characterization of 10 BOE determinants, supplying an empirical platform for stage-targeted interventions.

The pandemic interval furnishes more granular intelligence than extant global aggregate surveys. Logistic dissection of the COVID-19 period isolates January holiday rotations, physician-intubation encounters and unexpectedly vulnerable senior staff as independent drivers, thereby refining the generic observation that “COVID-19 increased risk.” The inversion of the traditional nurse-over-doctor exposure gradient underscores the context-specificity of occupational hierarchies during aerosol-generating procedures and supplements the literature dominated by pre-COVID nursing samples (14, 17).

Finally, the measurable success of the XS-glove pilot and the association between readily available hepatitis-B serology and higher reporting rates move the discussion from aspirational guidelines (10, 23) to demonstrably implementable controls. Department-specific dissection of instrument-related injuries (neurosurgical drills, cardiovascular catheters, endoscopic forceps) and prospective evaluation of the proposed holiday-drill and senior-staff refresher modules constitute the evidentiary next step.

## 5 Conclusion

This 11-year longitudinal audit demonstrates that the epidemiology of blood-borne occupational exposures (BOEs) is neither static nor solely governed by individual competence; it is repeatedly reshaped by calendar effects, departmental case-mix and, most dramatically, by public-health emergencies. The pandemic-induced inversion of the traditional nurse-over-doctor risk gradient, the January holiday peak driven by rotational staffing, and the unexpected vulnerability of mid-career clinicians all illustrate how rapidly the risk landscape can be redrawn. Sustained control therefore requires prevention frameworks that are equally dynamic: seasonally calibrated drills, role-specific protective devices for physicians, and a provincial registry that follows every temporarily deployed employee. Embedding these measures, together with pre-placement simulation training and universal verification of hepatitis-B immunity into routine quality-management cycles will narrow the remaining gap between incident and reported exposure and, ultimately, eliminate the great majority of preventable sharps injuries in Chinese tertiary care and comparable settings worldwide.

## Data Availability

The original contributions presented in the study are included in the article/Supplementary material, further inquiries can be directed to the corresponding author.
