# Supplementary material for: Trends and risk factors of bloodborne occupational exposure among healthcare workers in a Chinese tertiary hospital (2012–2022)
Source: Front Public Health. 2025 Oct 8;13:1619355. doi: 10.3389/fpubh.2025.1619355 (PMC12540332; doi:10.3389/fpubh.2025.1619355)
Supplement: Supplementary file 1 [file Table_1.DOCX]

**Supplementary Table 1 Staff composition and occupational composition from 2012-2022**

| Staff composition | | | 2012 year | | 2013 year | | 2014 year | | 2015 year | | 2016 year | | 2017 year | | 2018 year | | 2019 year | | 2020 year | | 2021 year | | 2022 year | | *χ^2^* | P |
| --- | --- | --- | --- | --- | --- | --- | --- | --- | --- | --- | --- | --- | --- | --- | --- | --- | --- | --- | --- | --- | --- | --- | --- | --- | --- | --- |
|  |  |  | A | B | A | B | A | B | A | B | A | B | A | B | A | B | A | B | A | B | A | B | A | B |  |  |
| H | C | | 15 | 20.55 | 8 | 9.30 | 14 | 12.39 | 26 | 18.31 | 13 | 7.03 | 26 | 12.26 | 30 | 12.61 | 39 | 16.88 | 14 | 10.69 | 26 | 15.95 | 23 | 15.23 | 62.91 | 0.00 |
|  | D | | 40 | 54.79 | 52 | 60.47 | 67 | 59.29 | 69 | 48.59 | 99 | 53.51 | 114 | 53.77 | 119 | 50.00 | 78 | 33.77 | 66 | 50.38 | 52 | 31.90 | 33 | 21.85 |  |  |
|  | E | | 1 | 1.37 | 0 | 0.00 | 1 | 0.88 | 2 | 1.41 | 2 | 1.08 | 6 | 2.83 | 2 | 0.84 | 6 | 2.60 | 3 | 2.29 | 7 | 4.29 | 6 | 3.97 |  |  |
|  | G | | 56 | 76.71 | 60 | 69.77 | 82 | 72.57 | 97 | 68.31 | 114 | 61.62 | 146 | 68.87 | 151 | 63.45 | 123 | 53.25 | 83 | 63.36 | 85 | 52.15 | 62 | 41.06 |  |  |
| I | J | C | 1 | 1.37 | 2 | 2.33 | 1 | 0.88 | 4 | 2.82 | 1 | 0.54 | 3 | 1.42 | 6 | 2.52 | 4 | 1.73 | 4 | 3.05 | 3 | 1.84 | 6 | 3.97 |  |  |
|  |  | D | 0 | 0.00 | 0 | 0.00 | 0 | 0.00 | 3 | 2.11 | 1 | 0.54 | 0 | 0.00 | 0 | 0.00 | 0 | 0.00 | 0 | 0.00 | 0 | 0.00 | 0 | 0.00 |  |  |
|  |  | G | 1 | 1.37 | 2 | 2.33 | 1 | 0.88 | 7 | 4.93 | 2 | 1.08 | 3 | 1.42 | 6 | 2.52 | 4 | 1.73 | 4 | 3.05 | 3 | 1.84 | 6 | 3.97 |  |  |
|  | K | C | 7 | 9.59 | 9 | 10.47 | 20 | 17.70 | 22 | 15.49 | 55 | 29.73 | 46 | 21.70 | 62 | 26.05 | 95 | 41.13 | 41 | 31.30 | 71 | 43.56 | 48 | 31.79 |  |  |
|  |  | D | 8 | 10.96 | 14 | 16.28 | 9 | 7.96 | 13 | 9.15 | 11 | 5.95 | 9 | 4.25 | 16 | 6.72 | 8 | 3.46 | 2 | 1.53 | 3 | 1.84 | 32 | 21.19 |  |  |
|  |  | E | 0 | 0.00 | 0 | 0.00 | 0 | 0.00 | 0 | 0.00 | 0 | 0.00 | 3 | 1.42 | 1 | 0.42 | 0 | 0.00 | 0 | 0.00 | 0 | 0.00 | 0 | 0.00 |  |  |
|  |  | G | 15 | 20.55 | 23 | 26.74 | 29 | 25.66 | 35 | 24.65 | 66 | 35.68 | 58 | 27.36 | 79 | 33.19 | 103 | 44.59 | 43 | 32.82 | 74 | 45.40 | 80 | 52.98 |  |  |
|  | F | | 1 | 1.37 | 1 | 1.16 | 1 | 0.88 | 3 | 2.11 | 3 | 1.62 | 5 | 2.36 | 2 | 0.84 | 1 | 0.43 | 1 | 0.76 | 1 | 0.61 | 3 | 1.99 |  |  |
|  | G | | 17 | 23.29 | 26 | 30.23 | 31 | 27.43 | 45 | 31.69 | 71 | 38.38 | 66 | 31.13 | 87 | 36.55 | 108 | 46.75 | 48 | 36.64 | 78 | 47.85 | 89 | 58.94 |  |  |
| G | | | 73 | 100.00 | 86 | 100.00 | 113 | 100.00 | 142 | 100.00 | 185 | 100.00 | 212 | 100.00 | 238 | 100.00 | 231 | 100.00 | 131 | 100.00 | 163 | 100.00 | 151 | 100.00 |  |  |

A:Number (people); B:Percentage (%)；C:Physicians；D:Nurses；E:Technicians；F:Non-medical personnel；

G:Total；H:Hospital staff; I:Non-hospital staff; J:Visiting Scholars; K:Students
